# Taxonomic and Environmental Variability in the Elemental Composition and Stoichiometry of Individual Dinoflagellate and Diatom Cells from the NW Mediterranean Sea

**DOI:** 10.1371/journal.pone.0154050

**Published:** 2016-04-25

**Authors:** Mariona Segura-Noguera, Dolors Blasco, José-Manuel Fortuño

**Affiliations:** Department of Marine Biology and Oceanography, Institut de Ciències del Mar (CSIC), Barcelona, Catalonia; National Taiwan Ocean University, TAIWAN

## Abstract

Here we present, for the first time, the elemental concentration, including C, N and O, of single phytoplankton cells collected from the sea. Plankton elemental concentration and stoichiometry are key variables in phytoplankton ecophysiology and ocean biogeochemistry, and are used to link cells and ecosystems. However, most field studies rely on bulk techniques that overestimate carbon and nitrogen because the samples include organic matter other than plankton organisms. Here we used X-ray microanalysis (XRMA), a technique that, unlike bulk analyses, gives simultaneous quotas of C, N, O, Mg, Si, P, and S, in single-cell organisms that can be collected directly from the sea. We analysed the elemental composition of dinoflagellates and diatoms (largely *Chaetoceros* spp.) collected from different sites of the Catalan coast (NW Mediterranean Sea). As expected, a lower C content is found in our cells compared to historical values of cultured cells. Our results indicate that, except for Si and O in diatoms, the mass of all elements is not a constant fraction of cell volume but rather decreases with increasing cell volume. Also, diatoms are significantly less dense in all the measured elements, except Si, compared to dinoflagellates. The N:P ratio of both groups is higher than the Redfield ratio, as it is the N:P nutrient ratio in deep NW Mediterranean Sea waters (N:P = 20–23). The results suggest that the P requirement is highest for bacterioplankton, followed by dinoflagellates, and lowest for diatoms, giving them a clear ecological advantage in P-limited environments like the Mediterranean Sea. Finally, the P concentration of cells of the same genera but growing under different nutrient conditions was the same, suggesting that the P quota of these cells is at a critical level. Our results indicate that XRMA is an accurate technique to determine single cell elemental quotas and derived conversion factors used to understand and model ocean biogeochemical cycles.

## Introduction

The C:N:P:Si ratio as well as nutrient quotas or concentrations in marine phytoplankton are routinely used in ocean biogeochemistry models to explain global patterns of plankton distribution and to predict primary production both qualitatively (in terms of elemental and biochemical composition) and quantitatively. Hence, these parameters are of critical importance to study, understand, model and predict ocean biogeochemical cycles [1, 2, 3]. Field studies have shown that these parameters may vary considerably in the ocean [4, 5]. Furthermore, experimental work has revealed taxonomic differences in macronutrient ratios in phytoplankton related to fundamental biochemical differences, or unique phenotypic strategies in response to their environment [6, 7, 8, 9, 10].

Several hypotheses have been put forward to explain the variability observed in the ocean, and to reconcile phytoplankton dynamics with the ratios of major nutrients in the water (e.g. [3, 11]). Unfortunately, data on the elemental composition of plankton in nature is still too sparse to validate these hypotheses [9, 12], specially for particulate phosphorus [13]. Two relatively new methods, energy dispersive X-ray microanalysis (XRMA, also abbreviated as EDX or EDS for energy dispersive X-ray spectroscopy) [14, 15] and synchrotron–based X-ray fluorescence microprobe (SXRF) [16], show promise to overcome this scarcity. However, these methods are not yet routinely applied and the existing data is still limited, available only for a few taxonomic groups and environmental conditions. Furthermore, very few studies have provided quantitative data (mass per unit volume), and used the same instruments and techniques to simultaneously measure all elements [17]. XRMA can overcome this problem, because, unlike other single-cell methods, it allows the simultaneous identification and quantification of all the elements (C, N, O, Na, Mg, Al, Si, P, S, Cl, K and Ca) present in the cell.

In this study, we have used XRMA to simultaneously determine the mass of C, N, O, Mg, Si, P and S in individual field marine dinoflagellate and diatom cells collected from different environments, in terms of nutrients availability and water column stratification, along the coast of the Catalan Sea (NW Mediterranean Sea). The species analysed during this study (*Prorocentrum* sp., *Dinophysis* spp., *Tripos* sp., *Protoperidinium* spp., *Rhizosolenia* sp., *Pseudo-nitzschia* sp. and *Chaetoceros* spp.) are among the most abundant species in the NW Mediterranean Sea [18, 19], and are all major components of the phytoplankton exported to the deep ocean [20]. This allowed us to compare the average stoichiometry of our cells with nutrients stoichiometry in deep NW Mediterranean Sea. Species assemblages varied accordingly from one site to another [21]. However, a few genera were present at different sites, which gave us the opportunity to observe the influence of the environment on the elemental composition of cells of the same species or genera.

To our knowledge, this is the first study that shows the elemental composition of single microphytoplankton cells collected from the field, including light elements (C, N and O). We focused on the inter-taxonomic variability of both the C:N:P ratios and the elemental concentration of these elements, as well as of O, Mg, Si and S. Our data indicate that environmental conditions affect the intracellular concentration of different elements independently, and therefore have an impact on the stoichiometry of both cells and populations.

## Methods

### Ethics Statement

No specific permits were required for the described field studies. No specific permissions were required for these locations/activities. The sampling sites are not privately-owned or protected, and the field studies did not involve endangered or protected species.

### Sampling sites and biogeochemical analyses

**Fig 1:**
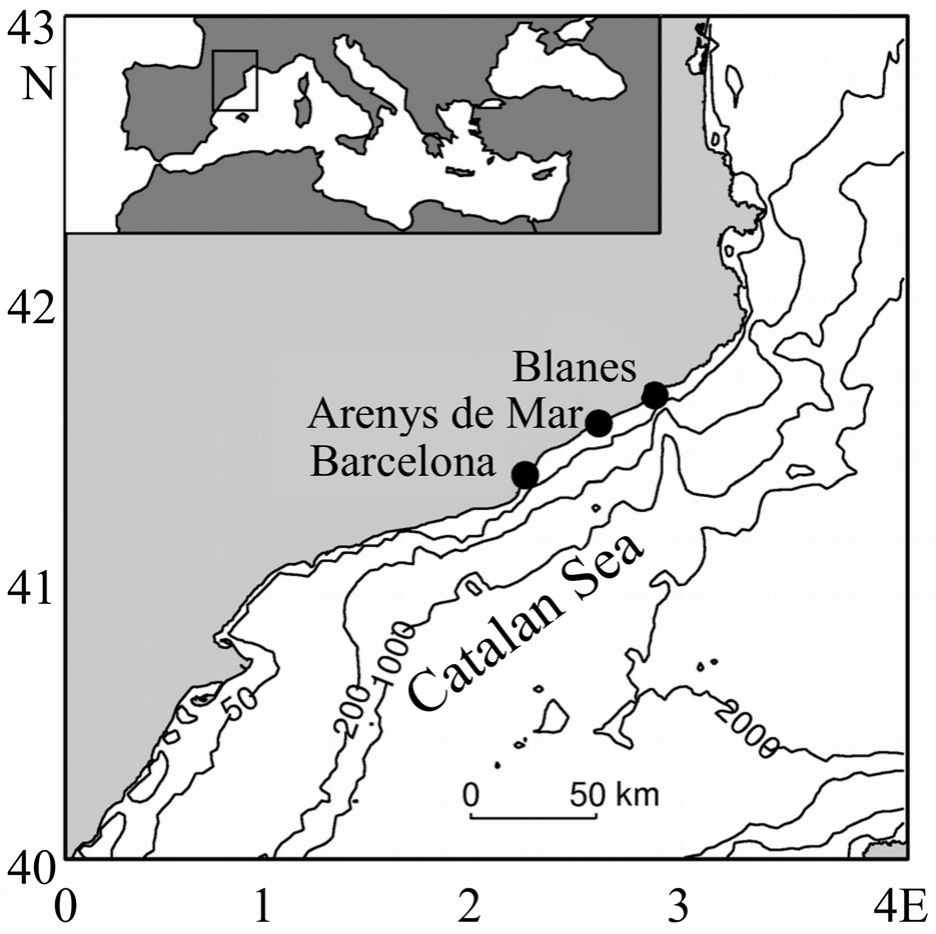
Map of the localization of the sampling sites at the Catalan coast. The inlet map shows the position of the Catalan Sea in the Mediterranean Sea.

Four surface water samples were taken at three different locations along the Catalan coast (Fig 1) with different environmental characteristics (Table 1).

**Table 1:** Physical and biogeochemical characteristics of the different sampling sites.

**Location**	Barcelona	Arenys de Mar	Blanes Bay	Barcelona
**Id. location**	CS M	Harbour	Bay	CS S
**Date**	26 January 2006	9 March 2006	16 May 2006	25 May 2006
**Position**	41°34’N, 2°32’E	41°34’N, 2°32’E	41°34’N, 2°32’E	41°34’N, 2°32’E
**Bottom depth (m)**	40	2	20–24	40
**Sampling depth (m)**	surface	surface	0.5	surface
**Distance from coast (m)**	2500	inside the harbour	800	2500
**Temperature (°C)**	12.6	12.3	18.4	19.0
**Salinity**	38.3	38.9	37.8	37.8
**Density (σ-T, Kg m**^**-3**^**)**	29.0	29.6	27.3	27.2
**Chlorophyll *a* (μg L**^**-1**^**)**	0.75	0.65	2.45	0.27
**Phosphate (μmol L**^**-1**^**)**	0.09	0.09	0.10	0.07
**Nitrate (μmol L**^**-1**^**)**	3.02	4.92	0.54	0.12
**Nitrite (μmol L**^**-1**^**)**	1.19	0.08	0.14	0.05
**Ammonium (μmol L**^**-1**^**)**	0.89	0.10	0.13	0.07
**Silicate (μmol L**^**-1**^**)**	1.81	3.07	0.13	0.22

CS M: Continental Shelf Mixed, H: Harbour, B: Bay, CS S: Continental Shelf Stratified.

The first sample was taken inside the harbour of Arenys de Mar (41°34’N, 2°32’E) during a routine sampling of the SEED program (GOCE-CT-2005-003875, EC). This 2-meter deep harbour is a good example of a heavily eutrophicated area in the Mediterranean, where red tides occur often. The sample (from now on “Harbour”) was taken at the end of the exponential growth phase of a bloom of the dinoflagellate *Alexandrium minutum* [22]. Nutrient concentrations (Table 1) were high compared to usual values observed in the Catalan Sea [23]. Most phytoplankton cells were dinoflagellates of the species *A*. *minutum*, *Scrippsiella* sp. and *Dinophysis* cf. *punctata*.

The second sample was taken at the Blanes Bay Microbial Observatory (BBMO, NW Mediterranean 41°40’N, 2°48’E, from now on “Bay”). The Blanes Bay is a well-studied (e.g. [24]) open oligotrophic bay located at the Catalan coast, and it is a good example of the oligotrophic Mediterranean coastal ecosystem, directly impacted by human and terrestrial influences from the nearby coast. The site is about 1 km offshore (41°40’N, 2°48’E) with a depth between 20 and 24 meters. The sample was taken in May, at the end of a typical spring bloom when chlorophyll *a* concentration was still high but nutrients were already low (Table 1). The microphytoplankton population was quite diverse, although dinoflagellates dominated, as is characteristic for this region during spring [25]. The cells analysed from this sample were dinoflagellates of the species *Tripos fusus*, *Tripos furca*, *D*. cf. *punctata*, *Dinophysis* cf. *acuta*, *Prorocentrum* cf. *micans* and *Protoperidinium* spp., and some diatoms of the genera *Chaetoceros* spp., *Pseudo-Nitzschia* spp. and *Rhizosolenia* sp.

The remaining two samples were both taken under contrasting environmental conditions on the continental shelf at the Coastal Monitoring Station of Barcelona (Estació Litoral de Barcelona, 41°53’N, 2°14’E). The sampling site is about 2.5 km offshore the city of Barcelona, on a 40 m deep water column. The first sample was taken in January 2006, under mixed water column conditions, and the second on May 2006, after the thermocline was already established. In January (from now on, “CS Mixed” for Continental Shelf Mixed site), the surface temperature as well as the nutrient and chlorophyll *a* concentration indicated typical Mediterranean well mixed winter conditions (Table 1). The microphytoplankton population was composed mostly by diatoms and nanoflagellates, as is typical for this time of the year [26]. The cells were diatoms of the genera *Chaetoceros* spp., *Pleurosigma* sp. and *Thalassiosira* spp. During the sampling in May (from now on, “CS Stratified” for Continental Shelf Stratified site), a clear vertical gradient of salinity, temperature and density was observed, with low nutrients and low chlorophyll *a* (Table 1). The microphytoplankton population present was a mixture of dinoflagellates and diatoms, all of them at very low concentrations. The cells from this sample were the genera *D*. cf. *punctata*, *P*. cf. *micans* and *Protoperidinium* spp., as well as diatoms of the genera *Chaetoceros* spp. and *Pseudo-nitzschia* spp.

Temperature, salinity, and other basic parameters such as chlorophyll *a* (Chla) concentration and dissolved inorganic nutrients (NO_3_^-^, NO_2_^-^, NH_4_^+^, PO_4_^3-^ and Si[OH]_4_) were measured at all sites (Table 1). A full description of the methods used to analyse those parameters at the Harbour, Bay, and Continental Shelf sites are given in Alonso-Sáez et al. [24], Van Lenning et al. [27] and Romero et al. [28], and Arin et al. [26], respectively.

### Elemental analysis of single cells

Water samples for XRMA analysis were collected in 5 to 8 L plastic bottles after pre-screening with a 200 μm nylon mesh to remove large mesozooplankton. A complete description of the methodology followed to collect, prepare, and analyse the elemental composition of microphytoplankton cells using XRMA can be found in Segura-Noguera et al. [15]. Briefly, the cells were concentrated onto a 20 μm nylon mesh and washed to remove the salt. After washing, the cells were centrifuged at 15°C over a 10 mm diameter Cu grid coated with formvar. The grid was air-dried and stored in a vacuum chamber until analysis. Cells were viewed and analysed for elements in a Hitachi S-3500N Scanning Electron Microscope (SEM), equipped with an energy-dispersive spectrometer Si(Li) detector (Bruker AXS). The detector processes X-rays with atomic number >2, thus including light elements, and has a resolution ≤ 129 eV (calibrated with Mn Kα lines). To avoid interference of holder material with sample elements, as well as with the X-ray spectra, the sample grid was mounted over a customized high-resolution holder (Hitachi) at a distance of 17 mm over the holder bottom [15]. With this modification, the thin-film analysis conditions of the Scanning Transmission Electron Microscope (STEM) were obtained in the SEM. The microscope was operated at 15 kV of electron beam energy, 15 mm of working distance, accumulation time of 100 sec (live time) per analysis, and 15° of tilt angle. The X-ray spectrum was recorded from an area that circumscribes the specimen [14]. The software used to acquire the spectra and to obtain the counts per second of each element present in the spectra was QUANTAX 1.6 (Bruker AXS).

Cells were identified by the imaging system, and two perpendicular axes of the cell were measured from the SEM pictures of the analysed cells using the software QUARTZ PCI 5.1. After, cell volumes were calculated following Sun and Liu [29], assuming depth to be half the width, except in *Protoperidinium* sp. and *Tripos* sp., for which depth was assumed to be equal to width.

Latex beads (Agar Scientific) were used to calibrate carbon, and the calibration constants for other elements were obtained following Norland et al. [14]. The detection limit of the method is 0.16% [15]. In the present study, only elemental quotas above the detection limit, which are more than 98% of the quotas measured, are shown and used to calculate ratios. The standard error of the elements quantification are 7–8% for C, O, Mg, P, S, Ca and 10–11% for N and K [15]. Total cellular dry weight per cell was calculated adding the molecular weight of all the quantified elements (C, N, O, Na, Mg, Al, Si, P, S, Cl, K and Ca), and assuming 1.6 mols of H per each mol of C (from equation 2 in Fraga [30]). Data of our single-cell analysis is shown in S1 Table.

### Statistical analyses

Element *vs*. volume regressions were studied first normalizing the variables by log transformation. Conversion factors to predict cellular carbon from cell volumes were estimated as the slope of the regression between element mass and volume, using Ordinary Least Squares (OLS) regression model type I [31, 32]. Elemental ratios can be estimated as the average of individual ratios in a given population, or as the slope of the linear regression of each element with P (e.g. [33, 34, 35]). The linear regression method minimizes the error between ratios when the concentrations are low [36], and so it is used in this study to compare ratios between phylogenetic groups. Both estimates are similar when the intercept of the regression is close to 0.

Element *vs*. element regressions were estimated using Standard Major Axis (SMA), a type II regression model that takes into account differences in the scale of both axes (e.g. C:P relationship). Extreme values were identified using the Quartile method (values smaller/higher than the first/third quartile minus/plus three times the interquartile range) and not used to calculate regression lines. The stoichiometries were first calculated independently for each group. Afterwards, tests for differences between y-intercepts and slopes of diatoms and dinoflagellates were performed. The software SMATR v2.0 [37] was used to calculate both OLS and SMA slopes and intercepts, along with *p*-values and confidence intervals, as well as differences and shifts in slopes between dinoflagellates and diatoms.

Inter- and intra-generic differences between elemental concentrations and stoichiometry were studied with the software PAST v3.06 [38]. Univariate non-parametric tests Mann-Whitney U was used in case of 2 groups, and Kruskal-Wallis for 3 groups. If differences were found, a Mann-Whitney pairwise comparison with Bonferroni corrected *p*-values was performed. Statistical significance was accepted when *p ≤* 0.01.

## Results and Discussion

### Single-cell *vs*. published elemental concentrations

**Fig 2:**
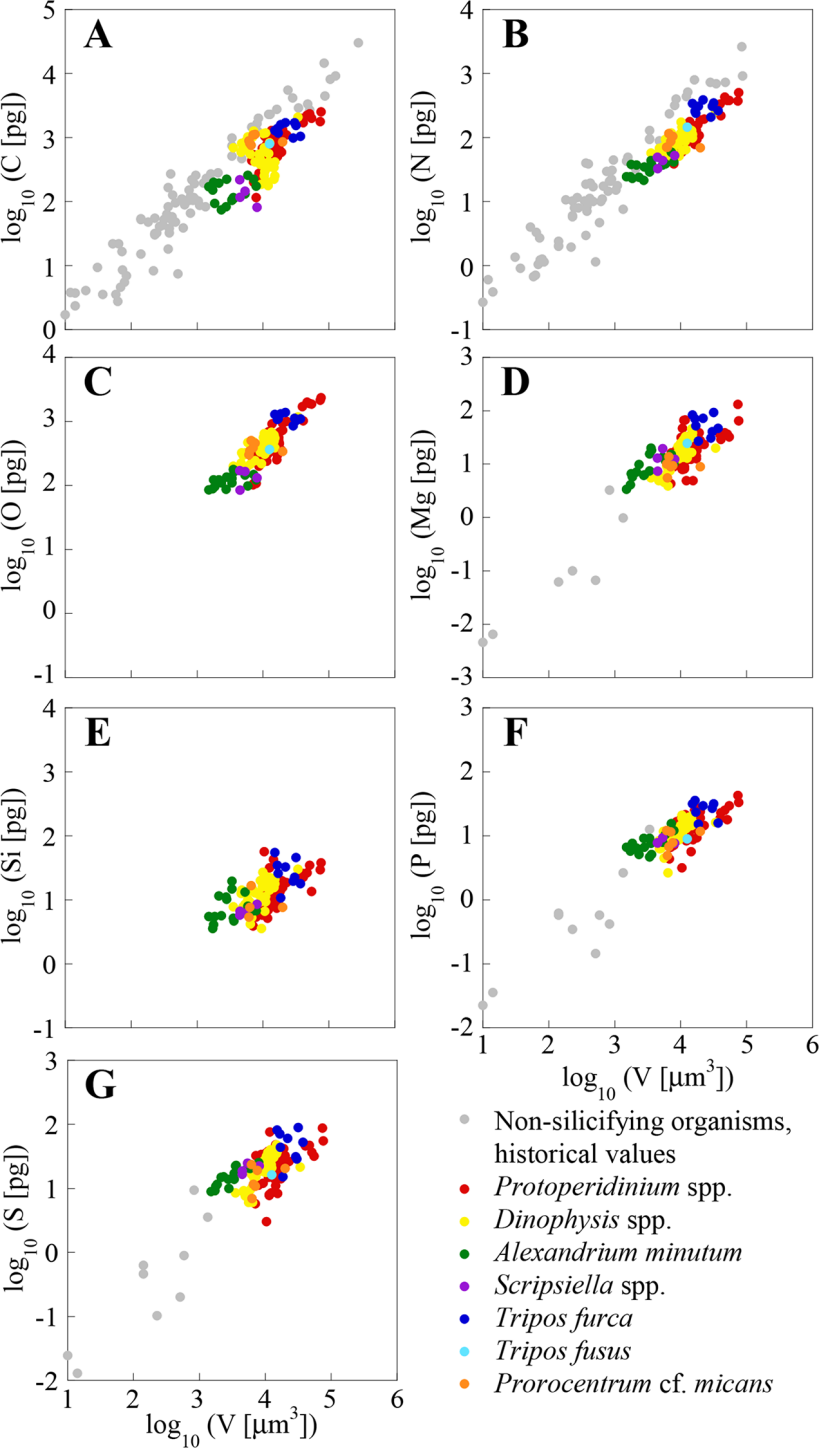
Log-log plots of the mass of C, N, O, Mg, Si, P and S (pg cell^-1^) *vs*. volume (μm^3^ cell^-1^). Dinoflagellate single-cell values from the NW Mediterranean Sea are presented along with historical values of cultured non-silicifying planktonic organisms obtained using bulk analysis techniques. Note that vertical axes vary between elements.

**Fig 3:**
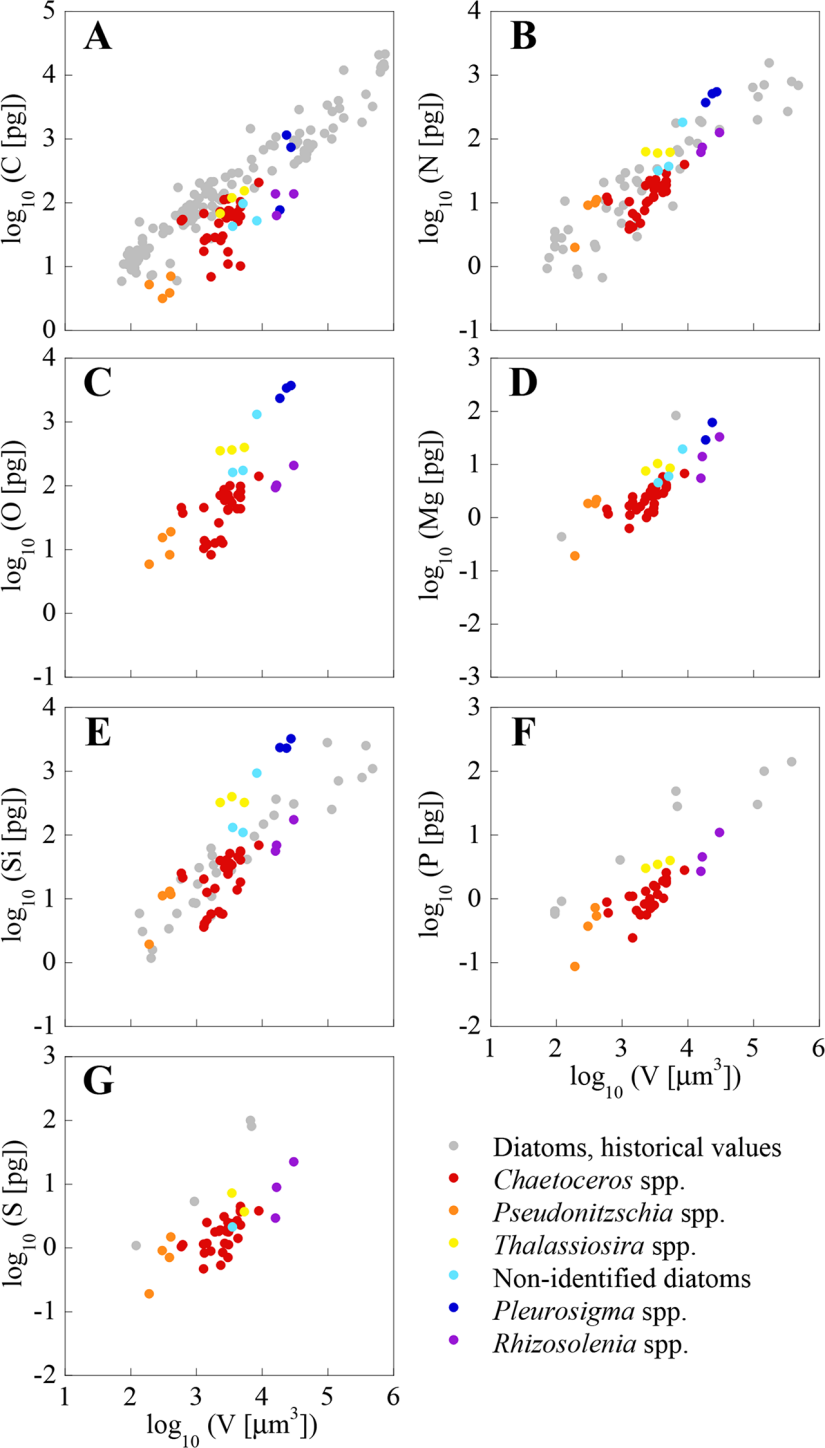
Log-log plots of the mass of C, N, O, Mg, Si, P and S (pg cell^-1^) *vs*. volume (μm^3^ cell^-1^). Diatom single-cell values from the NW Mediterranean Sea are presented along with historical values of cultured diatom cells obtained using bulk analysis techniques. Note that vertical axes vary between elements.

The elemental concentrations of our single-cell analyses, along with data from cultured cells of the same or close genera obtained with bulk analysis methods, are shown in Table 2. Our C, N and P concentrations are within the ranges of published data for cultures of the same or close genera, although, mostly, fall at the low end of these ranges. This can also be observed in Figs 2 and 3 for dinoflagellates and diatoms, respectively, which include all our cells and additional genera from the literature. The average mass of C, N, O, Si, P, and S per unit volume for the cells of the same species or genera, and of the same site are shown in S2 Table, which also includes the mean cell size and dry weight.

**Table 2:** Comparison between the elemental concentration (fg μm^-3^) of diatoms and dinoflagellates of the same or close genera analysed with bulk analysis techniques (CHN and HR-ICPMS) and single-cell methods (NMP and XRMA). Single-cell data presented in this table corresponds to the range of individual measurements of cells of the same genera.

Species	method	C	N	O	Mg	Si	P	S	K	Ca	References
**Dinoflagellates**											
***Alexandrium catenella***	CHN	177.8	38.9								[32]
***Alexandrium minutum***	XRMA	21.9–113.3	5.3–21.5	15.6–69.5	1.3–5.6	0.8–5.8	1.4–4.3	3.1–6.9	2.9–10.8	0.1–0.5	This study
***Scrippsiella* sp.**	CHN	49.0–2373	7.0–47.1								[32, 39]
***Scrippsiella* sp.**	XRMA	26.0–49.5	7.3–11.0	18.8–38.0	1.6–3.6	1.2–1.5	1.7	3.7–4.6	2.5–4.8	0.2–0.4	This study
***Dinophysis* sp.**	NMP	66.8–145.9	1.7–11.1				0.4–1.4				[40]
***Dinophysis* sp.**	CHN	222.9	13.0				1.8				[40]
***Dinophysis* cf. *acuta***	XRMA	62.6	7.6	35.2	0.6	0.9	0.5	0.6	0.6	0.0	This study
***Dinophysis* cf. *punctata***	XRMA	14.7–193.0	3.6–16.9	14.6–79.6	0.5–3.0	nd– 3.0	0.4–2.2	0.8–3.4	0.3–5.1	0.04–1.2	This study
***Tripos fusus***	CHN	69.7	15.3								[32]
***Tripos fusus***	XRMA	62.2	11.3	28.4	2.0	nd	0.7	1.3	nd	0.2	This study
***Tripos furca***	XRMA	28.0–84.8	7.0–22.1	29.4–84.5	1.1–5.4	0.5–3.6	0.4–2.2	0.8–5.3	0.5–3.6	0.1–4.0	This study
***Protoperidinium* spp.**	CHN	53.6–228.8									[32]
***Protoperidinium* spp.**	XRMA	5.4–159.9	2.4–13.0	1.3–66.0	0.3–5.9	nd– 5.4	0.1–2.8	0.2–6.5	0.1–5.2	nd– 3.0	This study
***Prorocentrum* spp.**	CHN	81.1–539.0	8.6–48.8					6.7			[8, 32, 41, 42, 43]
***Prorocentrum minimum***	HR-ICPMS				3.9		0.5	11.2	8.2	0.0	[8]
***Prorocentrum* cf. *micans***	XRMA	42.8–157.8	3.5–17.2	16.9–74.6	0.4–2.1	nd– 2.5	0.6–1.9	1.0–3.8	0.8–2.3	0.4–1.1	This study
**Diatoms**											
***Chaetoceros* spp.**	CHN	35.6–202.8	4.9–41.4			8.4–35.8					[44, 45, 46, 43, 47]
***Chaetoceros* spp.**	XRMA	2.2–89.5	2.5–20.8	5.0–78.5	0.4–2.5	2.3–42.8	0.2–1.5	0.2–1.8	0.2–2.2	0.03–2.7	This study
***Nitzschia* spp.**	CHN	34.6–132.1	1.8–23.9			6.5–7.3					[8, 44, 47]
***Nitzschia brevirostris***	HR-ICPMS				3.7		7.7	9.3	23.9	2.7	[8]
***Pseudo*-*nitzschia* sp.**	XRMA	27.3	10.3	30.6	1.0	10.2	0.4	1.0	0.4	nd	This study
***Rhizosolenia* spp.**	CHN	6.7–62.1	1.4			2.3					[44, 48]
***Rhizosolenia* sp.**	XRMA	8.8	3.9	6.0	0.3	3.6	0.2	0.2	nd	0.04	This study
***Thalassiosira* spp.**	CHN	22.0–370.9	3.9–78.3			9.8–43.4		0.7–2.2			[44, 49, 43, 47, 48, 50, 39, 51]
***Thalassiosira* spp.**	HR-ICPMS				12.6		4.3–7.4	5.8–15.1	28.2–30.9	2.2–6.4	[8]
***Thalassiosira* spp.**	XRMA	28.6–35.2	22.5–27.5	5.5–11.4	1.6–3.3	60.1–141.6	0.7–1.3	0.7–2.1	0.8–1.8	0.2–0.6	This study

CHN: Carbon+Hydrogen+Nitrogen elemental analysis; XRMA: X-Ray Microanalysis; NMP: Nuclear Microprobe; HR-ICPMS: High-Resolution Inductively Coupled Plasma Mass Spectrometry. *n* = number of cells. nd = not detected.

Relatively lower concentrations compared to published values were expected for two reasons: first, we used a single-cell method that excluded from the analysis extraneous material, like dead cells, detritus, or other organic extracellular material in the analysis, which are particularly rich in C and N [52]. The extracellular material significantly changes the intercepts but not the slopes of C and N mass *vs*. volume compared to published data [15]. And second, in contrast to cultured cells, which are usually harvested on exponential phase, our cells collected from the field had experienced different environmental histories and could be at any growth phase, even within the same sample. This variability translates into different biochemical compositions and hence elemental concentrations (e.g. [4]).

Low C concentrations are unlikely to be the result of methodological constraints: historically, XRMA has been deemed unsuitable for microphytoplankton cells because the calculated interaction volume (that is, the volume of sample where X-rays are generated) is smaller than the theoretical cell thickness (at least 20 μm). However, diatoms and dinoflagellates, with different thicknesses and densities, can be overpenetrated by a 15 kV electron beam as a result of drying the cells during SEM sample preparation [15]. All the cells analysed had a mass to area ratio below the most restrictive theoretical limits of beam penetration (4.8 and 5.7 pg μm-^2^ for dinoflagellates and diatoms, respectively [15]). Finally, the completeness of the beam penetration into the largest diatoms was also verified by the good agreement of the Si concentrations with data of Brzezinski [44] (Table 2, Fig 3E).

Some of our single-cell C concentrations, mostly in diatoms, were even lower than the published values: highly silicified diatom cells from the CS Mixed sampling site in January (mostly *Thalassiosira* and *Pleurosigma*, but also some *Chaetoceros*), as well as thin diatoms from the Bay in May (*Pseudo-nitzschia* and *Rhizosolenia*). Some of these diatoms also had a P content below detection limit, which suggests us that they were not viable at the moment of sampling (two *Chaetoceros* cells, all *Pleurosigma* and the diatoms not identified). *Thalassiosira* cells, on the other hand, had an unusually high N and O content (Fig 3B and 3C), which suggests that those cells could had been storing nitrate.

The concentrations of Mg, K and Ca in the cells analysed are also lower, although of the same order of magnitude, than in cells of the same genera analysed using high-resolution induced coupled plasma mass spectrometry (HR-ICPMS, [8]) (Table 2). Our data is within the range of published S concentrations [53, 49, 41, 54, 17, 8], although again at the low end. However, our S and O results for dinoflagellates (2.33 ± 1.43 fg μm^-3^ and 40.12 ± 14.49 fg μm^-3^, respectively) and diatoms (O = 37.46 ± 38.43 fg μm^-3^) are comparable with other XRMA data of *Prochlorococcus* (2.61 ± 0.92 fg μm^-3^ and 40.65 ± 3.96 fg μm^-3^, respectively) and *Synechocococcus* (2.47 ± 0.70 fg μm^-3^ and 34.06 ± 9.73 fg μm^-3^, respectively) [17]. The discrepancy in S concentration could be due to the probable loss of S associated with dimethylsulfoniopropionate (DMSP) during the drying of the samples [41], a process that would leave only the S of the cell structural compounds.

Figs 2 and 3 show a large variability in both bulk analysis published values and our single-cell analysis. This is likely the consequence of differences in cell size, as well as of genotypic, environmental and methodological differences. The variability in the elemental concentration between cells of the same genera, and from the same sample (e.g., *A*. *minutum*, *T*. *furca*) is larger than the error of the analysis, as found by other authors using XRMA of individual organisms from the field [54, 55]. As pointed out by these authors, this variability is probably due to differences in life cycle and growth status between individual cells, which in field populations are most certainly not synchronized. Other researchers using the same or other single-cell analysis techniques to measure C, N, P, and S, have observed the existence of intra-population variability in clonal cultures [17, 56].

### Differences in the elemental concentration between diatoms and dinoflagellates

We explored the differences in elemental concentration among elements and between plankton groups by comparing the slopes of the OLS regressions of the single-cell measurements (Figs 2 and 3). All element *vs*. volume slopes, shown in Table 3, are significantly different from 0 (*p* ≤ 0.001). No significant differences in element *vs*. volume slopes were found between diatoms and dinoflagellates, except for Si (*p* ≤ 0.01). Menden-Deuer and Lessard [32], using published data available at the time, exhaustively examined C per volume relationships in plankton, concluding that dinoflagellates are significantly C denser than diatoms. Our results show them to be denser also in N, O, Mg, P and S, since there are significant elevation shifts between groups for all these elements (*p* ≤ 0.001). Were these results extrapolated beyond the limited set of species and cell volumes analysed in the present study (between 2000 and 40000 μm^3^), important errors in the prediction of C, N, O, P and S from volume measurements may occur when the same conversion equations are used for both groups.

**Table 3:** Results of the ordinary least squares regression of log_10_ transformed element concentration (pg cell^-1^) *vs*. log_10_ transformed volume (μm^3^ cell^-1^) of diatoms and dinoflagellates from the Catalan Sea.

Variable	Group	Log a	Low CI	Upp CI	b	Low CI	Upp CI	s_b_	*r*^2^	n	Common slope	*p*	Intercept of common slope
**Dryweight**	Diatoms	-1.002	-1.776	-0.227	0.961	0.740	1.182	0.110	0.630	47	0.819	0.143	-0.509
	Dinoflagellates	-0.073	-0.454	0.309	0.787	0.693	0.882	0.048	0.655	145			-0.199
**C**	Diatoms	-1.039	-1.767	-0.311	0.786	0.579	0.994	0.102	0.563	47	0.787	0.998	-1.041
	Dinoflagellates	-0.415	-0.964	0.135	0.787	0.651	0.923	0.069	0.477	145			-0.414
**N**	Diatoms	-1.128	-1.778	-0.477	0.693	0.504	0.883	0.085	0.566	44	0.792	0.267	-1.463
	Dinoflagellates	-1.263	-1.550	-0.975	0.809	0.737	0.880	0.036	0.779	145			-1.194
**O**	Diatoms	-1.763	-2.691	-0.834	1.034	0.769	1.299	0.125	0.578	47	0.902	0.294	-1.307
	Dinoflagellates	-0.948	-1.303	-0.592	0.881	0.793	0.969	0.045	0.734	144			-1.033
**Mg**	Diatoms	-2.222	-2.826	-1.619	0.793	0.619	0.966	0.083	0.658	46	0.730	0.386	-2.005
	Dinoflagellates	-1.614	-2.101	-1.128	0.702	0.581	0.822	0.061	0.485	143			-1.727
**Si**	Diatoms	-2.198	-3.290	-1.106	1.094	0.782	1.406	0.147	0.526	47			
	Dinoflagellates	-0.827	-1.271	-0.383	0.473	0.363	0.583	0.056	0.342	141			
**P**	Diatoms	-2.219	-2.776	-1.662	0.676	0.513	0.838	0.080	0.678	37	0.541	0.063	-1.761
	Dinoflagellates	-0.958	-1.279	-0.636	0.508	0.428	0.587	0.040	0.527	144			-1.092
**S**	Diatoms	-1.888	-2.469	-1.306	0.632	0.462	0.802	0.084	0.592	41	0.540	0.202	-1.576
	Dinoflagellates	-0.705	-1.158	-0.253	0.503	0.391	0.615	0.057	0.354	145			-0.855

Shown in this table are the y-intercept (log a) and the slope (b) of the regression equations, the interval of confidence (Low CI and Upp CI), the standard error of the slope (*s*_b_), the coefficient of determination (*r*^2^), the number of data points included (n), the common slope (when there are no significant differences between the slope of both groups), its *p*-value, and the intercept of each group with the common slope. The elemental quota can be determined from volume based on the equation log_10_ element (pg cell^-1^) = log a + b x log_10_ volume (μm^3^). Same element slopes of diatoms and dinoflagellates are not statistically different, except for Si (*p* = 0.002). The intercepts of the dinoflagellates regression lines are larger than those of diatoms for the same element, except for N (*p* ≤ 0.001). However, this difference disappears when the common slope is calculated.

All elements, except O and Si in diatoms, had volume-scaling factors (i.e. slopes) significantly lower than 1.0 (*p* ≤ 0.001, Table 3). This indicates that the masses of C, N, Mg, P or S, as well as O and Si in dinoflagellates, are not a constant fraction of cell volume, but rather decrease with increasing cell volume. Thus, small cells have higher elemental content per volume than large cells. Slopes for C, N, O, Mg, as well as Si in diatoms, were not statistically different (*p* > 0.05). C and N slopes of our dinoflagellates were close to values compiled by Menden-Deuer and Lessard [32] (0.82 and 0.85, for C and N). Interestingly, P and S slopes, as well as Si in dinoflagellates, were significantly lower than the slopes for other elements both in diatoms and dinoflagellates (*p* ≤ 0.05), indicating that the size effect on P and S is important in these organisms.

Thingstad et al. [57] hypothesized that some osmotrophic microorganisms may get a competitive advantage by using a non-limiting nutrient to increase size, and thus reduce predation stress without increasing nutrient requirements. This is the strategy of diatoms, which have frustules conformed by elements with slopes larger than 1 (Table 3). However, the fact that all elements, except O and Si in diatoms, had slopes lower than 1, suggests that decreases in the concentration of an element are not always offset by increases in another element. Thus, these decreases could be achieved through an increase in water cell content, either by increasing cytoplasm volume or by enlarging vacuoles [58]. In such case, decreases in element content with increasing cell size will not necessarily lead to stoichiometric changes. Our results suggests that this “dilution effect” may be a possible mechanism for dinoflagellates to mitigate, to a certain point, the negative effect that the increase in size has on nutrient uptake.

The average P concentration for bacterioplankton, *Prochlorococcus* and *Synechococcus* is 2.93–3.17, 2.92 and 3.85 fg μm^-3^ respectively (calculated from data in [54, 59, 17]). The average P concentration in dinoflagellates and diatoms from our own results is 1.30 ± 0.68 and 0.58 ± 0.41 fg μm^-3^ respectively. This indicates that the P requirement is highest for bacterioplankton, followed by dinoflagellates, and lowest for diatoms. Low P quotas represent a clear ecological advantage in P-limited environments like the Mediterranean Sea.

The analysed diatoms have around 40–60% less C, P and S concentration than dinoflagellates, but N and O concentrations are only around 20% lower. A relatively low C concentration in diatoms with respect to other plankton groups has been previously observed [60, 32] and it has been linked to the presence of large intracellular vacuoles [58]. However, the presence of vacuoles does not explain why N is not proportionally reduced, unless N is stored inside them as nitrate (e.g. [43]). Such hypothesis is consistent with the observation that N concentration in diatoms collected in the nutrient-rich environment (CS Mixed) is higher than in the relatively low-nutrient environment (Bay). Additionally, the observed differences in C concentration between both groups could be due to the contribution of the carbohydrate theca of dinoflagellates. On the other hand, observed differences in P could be explained by a larger amount of DNA in the dinoflagellate nucleus compared to other eukaryotic cells (2.2–200 pg per nucleus [61]).

Finally, it should be noted that the extrapolation to other sizes or groups of the results presented here can be misleading. For example, extrapolating the C to V regression for dinoflagellates in Table 3 to picophytoplankton volumes yields lower values than those obtained for *Prochlorococcus* spp. (138–290 fg μm^-3^) and *Synechococcus* spp. (136–280 fg μm^-3^) by Heldal et al. [17] using XRMA. Because these analyses were done on cultured cells, it is difficult to determine whether these differences are a size effect. This points to the necessity of further analyses on single cells from field samples, covering a larger range of species, sizes and environmental conditions. However, till this data is gathered, when C or N are used as a proxy for cellular biomass in models and predictions, the confidence intervals of the equations being used should be explicitly stated, and the possible effect of errors should be considered in both the results and the conclusions.

### Elemental stoichiometry

The elemental composition of plankton can be expressed as the proportion of each element to total dry weight (percentage) or to one element. Both ratios are independent of the cellular size or mass and therefore allow for direct comparisons among species, plankton groups and ecosystems [62, 63]. However, they do not provide information on which element is controlling any observed change. Although P normalized numbers are most often used by oceanographers to quantitatively link the marine nutrient cycles in numerous biogeochemical applications, empirical and theoretical studies, as well as our individual cell values (S3 Table) show that elemental ratios vary greatly with taxa and growth conditions [4, 8].

**Fig 4:**
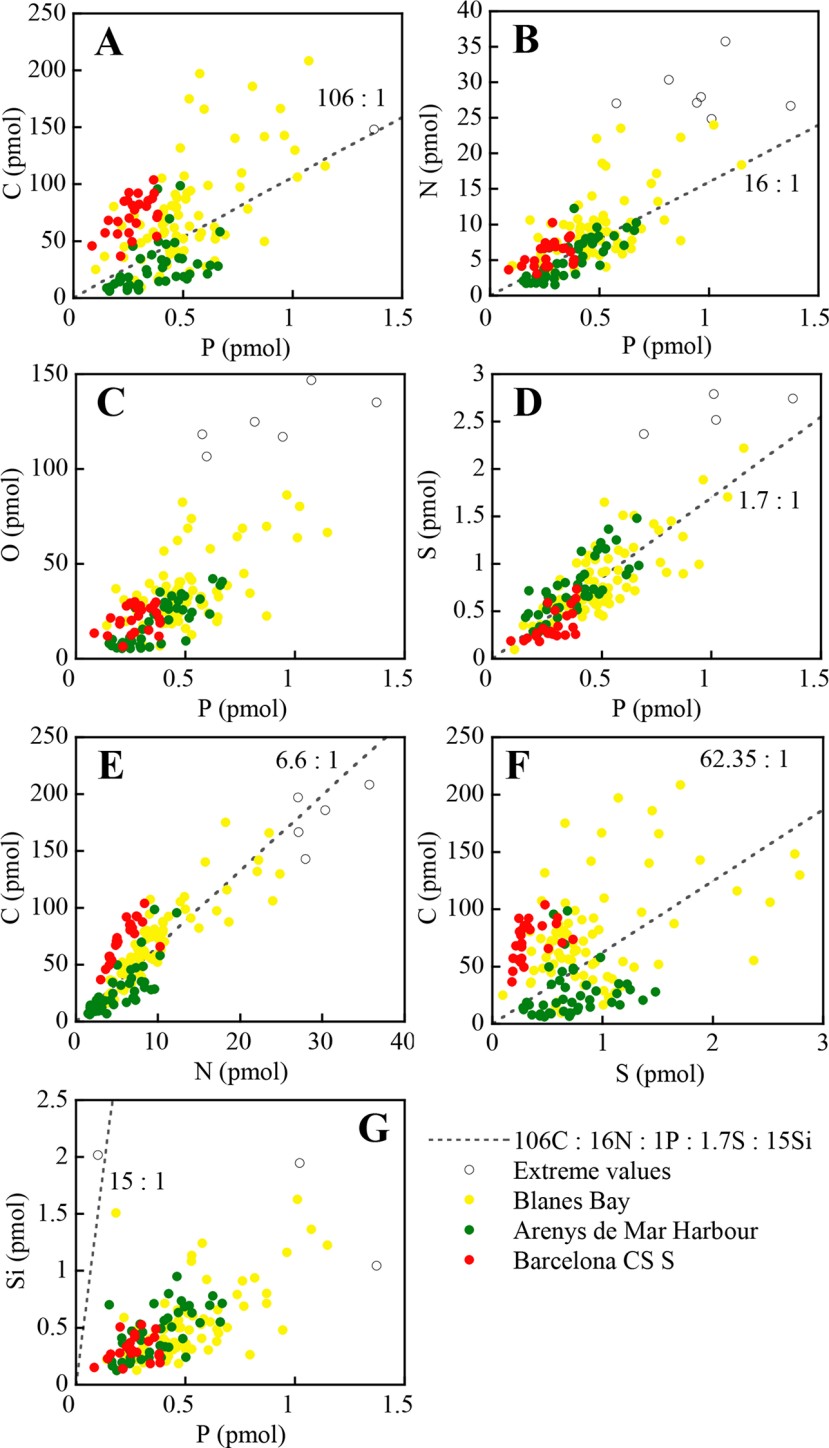
Molar relationships between C, N, O and S *vs*. P, C *vs*. N and S, and Si *vs*. P of individual dinoflagellates from the NW Catalan Sea. Cells from the same location are plotted with the same colour. Note that axes change.

**Fig 5:**
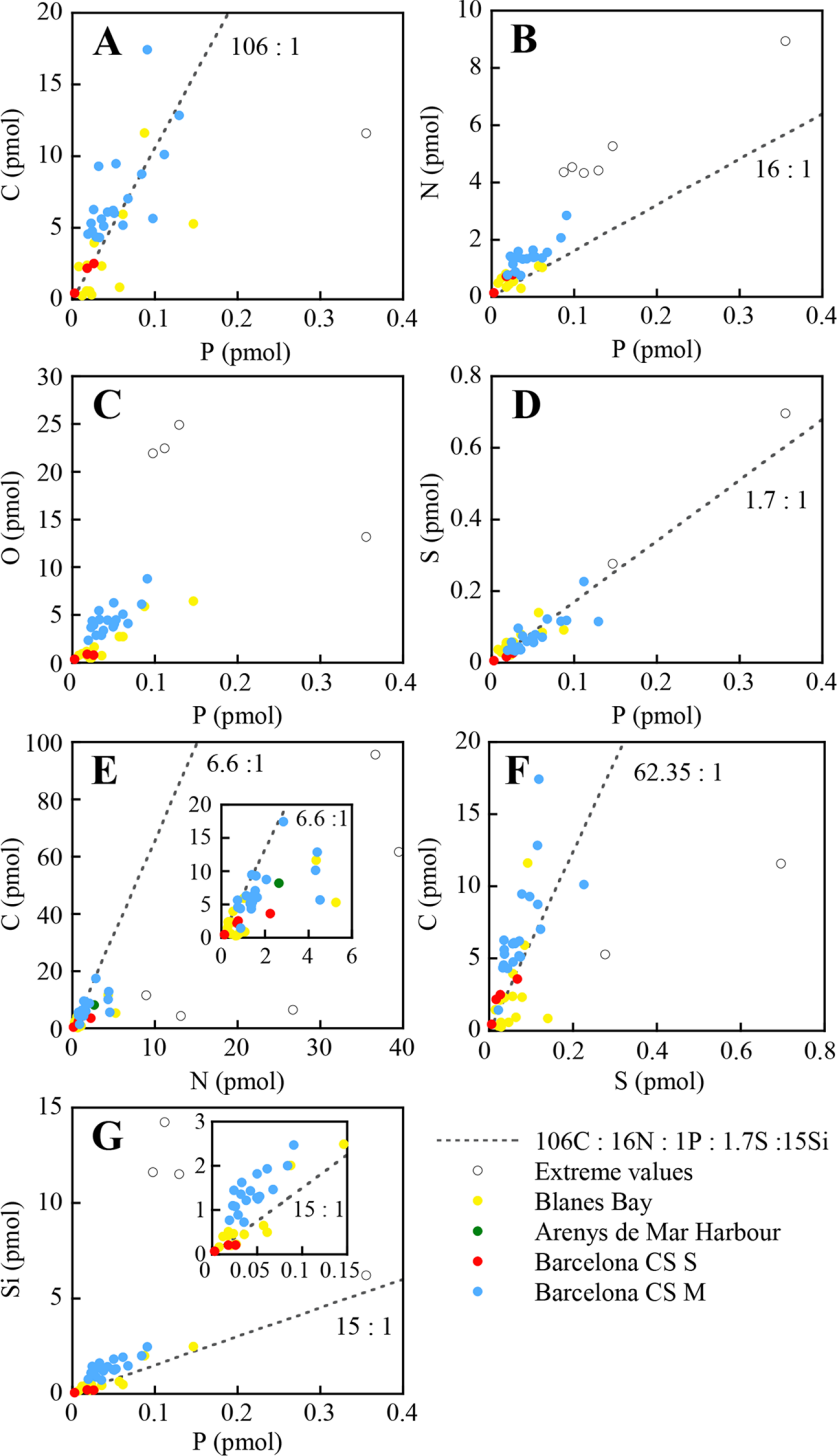
Molar relationships between C, N, O and S *vs*. P, C *vs*. N and S, and Si *vs*. P of individual diatoms from the NW Catalan Sea. Cells from the same location are plotted with the same colour. Note that axes change.

Individual values of dinoflagellate and diatom cells, with the cells grouped by sampling site, are shown in Figs 4 and 5, respectively. Slopes and intercepts of the regression lines (SMA) for dinoflagellates and diatoms are shown in Table 4. Significant differences (*p* ≤ 0.01) between diatoms and dinoflagellates were found for all slopes, except O:P and C:S. Note the tight relation that exists in the O:Si regression line (*r*^2^ = 0.92), which is reflecting the composition of the frustules. Based on the slope of the best fit regression lines, the overall elemental ratio C:N:O:P:S of the analysed dinoflagellates was 202.3±14.2: 24.6±1.5: 89.1±5.6: 1: 2.1±0.1 (slope ± standard error). The C:N:O:P:S:Si for diatoms was 109.8±14.1: 38.9±3.0: 73.53±8.8: 1: 1.5±0.2: 23.6±2.6.

**Table 4:** Results of the least square regression of different molar ratios (element *vs*. element) of single phytoplankton cells.

Ratio	Group	intercept	Low CI	Upp CI	slope	Low CI	Upp CI	s_b_	*r*^2^	*n*
**C:P**	Diatoms	0.12	-1.61	1.85	109.8	84.8	142.2	14.13	0.437	36
	Dinoflagellates	-27.09	-41.13	-13.05	202.3	1675.8	232.8	14.23	0.289	142
**N:P**	Diatoms	-0.27	-0.59	0.05	38.68	33.61	44.51	2.99	0.836	36
	Dinoflagellates	-1.72	-2.76	-0.68	24.57	22.29	27.08	1.53	0.489	137
**O:P**	Diatoms	0.20	-0.72	1.13	73.52	57.71	93.67	8.82	0.554	33
	Dinoflagellates	-9.74	-15.00	-4.48	89.10	78.61	100.99	5.59	0.456	137
**Mg:P**	Diatoms	-0.01	-0.04	0.01	3.42	3.01	3.90	0.23	0.859	36
	Dinoflagellates	-0.38	-0.53	-0.23	2.70	2.40	3.05	0.16	0.509	135
**Si:P**	Diatoms	0.05	-0.22	0.32	23.63	18.91	29.54	2.61	0.623	33
	Dinoflagellates	-0.13	-0.22	-0.04	1.41	1.23	1.61	0.10	0.376	137
**S:P**	Diatoms	0.00	-0.01	0.02	1.48	1.21	1.81	0.15	0.677	34
	Dinoflagellates	-0.19	-0.30	-0.09	2.18	1.97	2.41	0.10	0.630	141
**C:N**	Diatoms	0.60	-0.83	2.03	2.89	2.28	3.67	0.35	0.429	42
	Dinoflagellates	-1.60	-8.45	5.25	7.51	6.80	8.30	0.38	0.650	139
**C:O**	Diatoms	0.32	-0.99	1.64	1.21	0.97	1.51	0.13	0.539	40
	Dinoflagellates	0.68	-6.35	7.72	2.00	1.80	2.19	0.11	0.611	138
**C:S**	Diatoms	-0.78	-2.61	1.06	89.88	69.42	116.37	11.59	0.385	39
	Dinoflagellates	-7.40	-20.96	6.16	90.22	77.07	105.63	8.92	0.096	143
**Si:N**	Diatoms	-0.10	-0.48	0.18	0.96	0.76	1.21	0.11	0.514	39
**Si:C**	Diatoms	-0.39	-0.97	0.20	0.35	0.26	0.45	0.05	0.35	40
**O:Si**	Diatoms	0.67	0.29	1.05	2.38	2.18	2.61	0.11	0.924	40

Shown are the y-intercept and the slope of the regression equations, the interval of confidence (Low CI and Upp CI), the standard error of the slope (*s*_b_), the coefficient of determination (*r*^2^), and the number of data points included (*n*). All slopes are significantly different from zero (*p* ≤ 0.001). C:S and O:P slopes of dinoflagellates and diatoms are not statistically different (*p* = 0.983 and *p* = 0.149, respectively).

The C:P slope for dinoflagellates, as well as the N:P slopes for both groups (Table 4), are higher than the canonical Redfield ratio (116 and 16), but within the ranges of published values [4, 17]. The C:P slope for diatoms is not statistically different from the Redfield ratio (*p* = 0.787). In our study, the only dinoflagellate C:P and N:P values below the Redfield ratio have been found in *A*. *minutum*, and *Scrippsiella* sp. cells collected from the Harbour site. Both dinoflagellates were in exponential growing phase [22] in a high nutrient environment (Table 1). Thus, the low C:P and N:P ratios were likely caused by the relatively high P concentration, as expected in actively growing cells with high contents in nucleic acids [4].

The average C:S values for diatoms and dinoflagellates are similar to assumed phytoplankton values [64, 65]. However, the low coefficient of determination in dinoflagellates (Table 4, see also Fig 4F), casts doubt on the use of C as conversion factor for S, or vice-versa.

Because it is not yet possible to measure O with direct analytical methods, O:P ratios and C:O ratios in phytoplankton have been rarely reported in the literature, and most often the numbers provided rely on indirect estimates [66]. Very low C:O ratios have been occasionally found both in dinoflagellates and diatoms (S3 Table), possibly reflecting a low C, rather that high O concentrations. Such low values contradict our knowledge of the average elemental composition and proportions of planktonic biomolecules [67, 68, 4]. All our cells with a C:O < 1 also had a C:N < 3.7, a value that corresponds to the average ratio of proteins, pigments and nucleic acids [69], which represent up to 85% of the cells under nutrient rich conditions. This suggests that these cells were not alive at the moment of sampling. Theoretical estimations of O:P ratios based on different plausible combinations of main metabolites present in plankton cells (42 ± 16, [70]) and marine particulate matter (49.2, [71]) are lower than our measurements, probably because the theoretical calculations assume a ratio of C:N:P of 106:16:1, while our empirical ratio is larger for both groups, as evidenced by the fact that the two species with lower C:N:P ratio (two thecate dinoflagellate cells from the Harbour sample) have O:P ratio = 32.8, which is closer to the ones in the literature. On the other hand, the S:P ratios of our dinoflagellate and diatom cells (1.5 and 2.2, respectively, Table 4) are close to the canonical S:P = 1.7 [67], to the average value of 1.71 ± 0.70 for cyanobacteria [17], and within the range of published values for dinoflagellates and diatoms [8, 65].

Interestingly, Si was detected in both groups. In diatoms this element is a well known constituent of the exoskeleton [72]. The presence of Si has already been reported in the dinoflagellates *Ceratium hirundinella* [55] and *Exuviaella* sp. [45]. Recently, Si has also been detected in *Synechococcus* in concentrations of the same order than our dinoflagellates and with similar Si:P values (1.54 ± 1.18, [73]). However, the physiological role of Si in these organisms is still unclear. The Si:P ratio of our diatoms (Table 4) is higher than the Redfield-Brezinski ratio of 15:1 (derived from [44]), and of that found in chemostat studies (5.4, 6.7 and 3.8 [46]; 5.9 ± 1.3 [74]). However, it is consistent with ratios reported for diatoms in the Equatorial Pacific [73], which also had a low P quota. The tight relationship between Si and P is remarkable considering the well known inter-specific variability in frustule thicknesses, and the high variability of the Si content per cell [44].

The C:N slope in dinoflagellates (7.5 ± 0.4, Table 4) is within the range of ratios reported in the literature [4], close to Redfield's original ratio of 6.6 [75], and similar to particulate matter values in the NW Mediterranean Sea (C:N = 6–7 [36, 76]). Higher values (8.2 to 10) have been reported for cyanobacteria in cultures using the same single-cell methodology [17], and the difference has been attributed to cyanobacteria being relatively denser in C than N, which would explain also the higher C:P reported for cyanobacteria. The N:P ratio of our dinoflagellates and diatoms is more variable than the C:N ratio, as previously reported [4]. In diatoms, the C:N slope (2.9 ± 0.4) is in the lower side of the broad range of C:N ratios reported by Sarthou et al. [74] (range = 2.7–29.7). Our results show that the diatoms are N enriched compared to C, but some highly enriched cells, which are however not detected as extreme values, force the slope towards such low value (Fig 5E). If cells with N quota values above 4 pmol are removed from the regression, remaining only *Chaetoceros* and *Pseudo-nitzschia* cells, the C:N ratio increases to 5.4 ± 0.6 (n = 31), which is within the range of 85% of the diatom species gathered by Sarthou et al. [74] (range = 5.0–9.7). This evidences the need of more single-cell analyses to define the range of C:N values in the nature. Interestingly, the N:P ratio drops from 38.7 to 26.5 when these same diatom cells with high N quota are removed from the analysis. This ratio is not significantly different than the one from dinoflagellates (*p* = 0.571), and the common slope is N:P = 25.1 (n = 168).

### Phytoplankton single-cell stoichiometry and Western Mediterranean Sea biogeochemistry

The Mediterranean Sea has long been known as a nutrient-depleted basin in which, in contrast with other oligotrophic oceanic regions, P is the main limiting nutrient (e.g. [77, 78]). The main inputs of macronutrients in the NW Mediterranean Sea are river and groundwater discharges, and atmospheric deposition. They provide a total nutrient budget in terms of N and P significantly higher than the 16:1 Redfield ratio [79, 80]. It is well known that nutrient supply sets an upper limit to the biological production, but planktonic organisms exert a tight control on the elemental distribution particularly in deeper layers [81] because the main source of nitrate, orthophosphate and orthosilicate in the deep sea waters is the remineralization of sinking biological material. The dissolved inorganic nitrate:phosphate ratio of deep Western Mediterranean waters is between 20–23, e.g. [82, 83]. This high ratio, compared to the Redfield ratio is still difficult to explain and has been the object of different hypotheses [33, 84]. Correspondingly, our cells also show a high N:P ratio. This is consistent with studies performed in the NW Mediterranean Sea, which also found higher than Redfield ratios in picoplanktonic cultured cells (N:P = 21.2–62.3, [85]) and detritic matter (N:P = 32, [86]), both studies using XRMA methods. Also, bulk analysis studies of particulate organic mater from surface waters of the Mediterranean Sea found again N:P ratios higher than Redfield's (N:P = 18.8–23.2, [35, 36]).

A high N:P ratio could be a result of either high N or low P content. The fact that the Si:P in the diatoms analysed (27.8 ± 3.4) is also high, while the Si:N ratio (1.06 ± 0.08) is similar to what has been reported for diatoms (e.g. Si:N = 1.12 ± 0.33, range: 0.5–1.5 [44]; Si:N = 0.8 ± 0.3 [74]), as well as for the Western Mediterranean Sea (e.g. Si:N = 1.13 [87]; Si:N = 1.0 [83]), suggests that the high ratio is due to a lower P cell content. Unfortunately, there are very few published data to compare P with (Figs 2F and 3F). The consistently high observed cell N:P ratios can be an adaptation to low P availability in Mediterranean surface waters. One possible mechanism of adaptation to phosphorus limitation is the use of non-phosphorus lipids [88]. Phospholipid substitutions appear to be an important biochemical mechanism for cyanobacteria and eukaryotic phytoplankton to maintain photosynthesis in environments where phosphorus is scarce [10]. In cyanobacteria the substitute is the non-phosphorus membrane lipid sulphoquinovosyldiacylglycerol (SQDG). In eukaryotes the substitute are the non-phosphorus ‘betaine’ lipids. A preferential synthesis of non-phospholipds has been observed in the P-deprived phytoplankton of the Adriatic Sea at the Western Mediterranean Sea [89].

Microphytoplankton cells, like diatoms and dinoflagellates, contribute significantly to the export of organic matter from surface waters (e.g. [36]). Although it is not possible to generalize from our limited dataset, the species analysed in the present study, *Prorocentrum* sp., *Dinophysis* spp., *Tripos* sp., *Protoperidinium* spp., *Rhizosolenia* sp., *Pseudo-nitzschia* sp. and *Chaetoceros* spp. are among the most abundant species in the NW Mediterranean Sea [18, 19], and are all major components of the phytoplankton exported to the deep ocean [20]. So we can assume that our data, although scarce, is representative of the phytoplankton of this area. Consequently, and following Redfield's theory [67], the remineralization of the cells with high N:P, like the ones measured in this study, should be the source of the high N:P ratio observed in the deeper waters of the Western Mediterranean sea. Further single-cell XRMA of phytoplankton, including other abundant phytoplankton groups such as coccolithophorids, are needed to validate this hypothesis.

### Phylogenetic and environmental differences in elemental composition and stoichiometry between genera

The evidence of phylogenetic differences between phytoplankton groups in elemental concentrations or quotas has gained a lot of attention in recent years [8, 90]. Our dataset allowed us to study inter-generic differences of cells sampled under the same environmental conditions, as well as intra-generic differences of cells from contrasting environments.

Inter-generic differences were found between the dinoflagellates (1) *A*. *minutum* and *D*. cf. *Punctata* from the Harbour sample; (2) *T*. *furca* and *Protoperidinium* spp. from the Bay site, the last genera grouped into two very different size classes (< 25000 μm^3^ and > 45000 μm^3^); and (3) *P*. *micans*, *D*. cf. *punctata* and *Protoperidinium* spp. in the CS Stratified sample.

**Fig 6:**
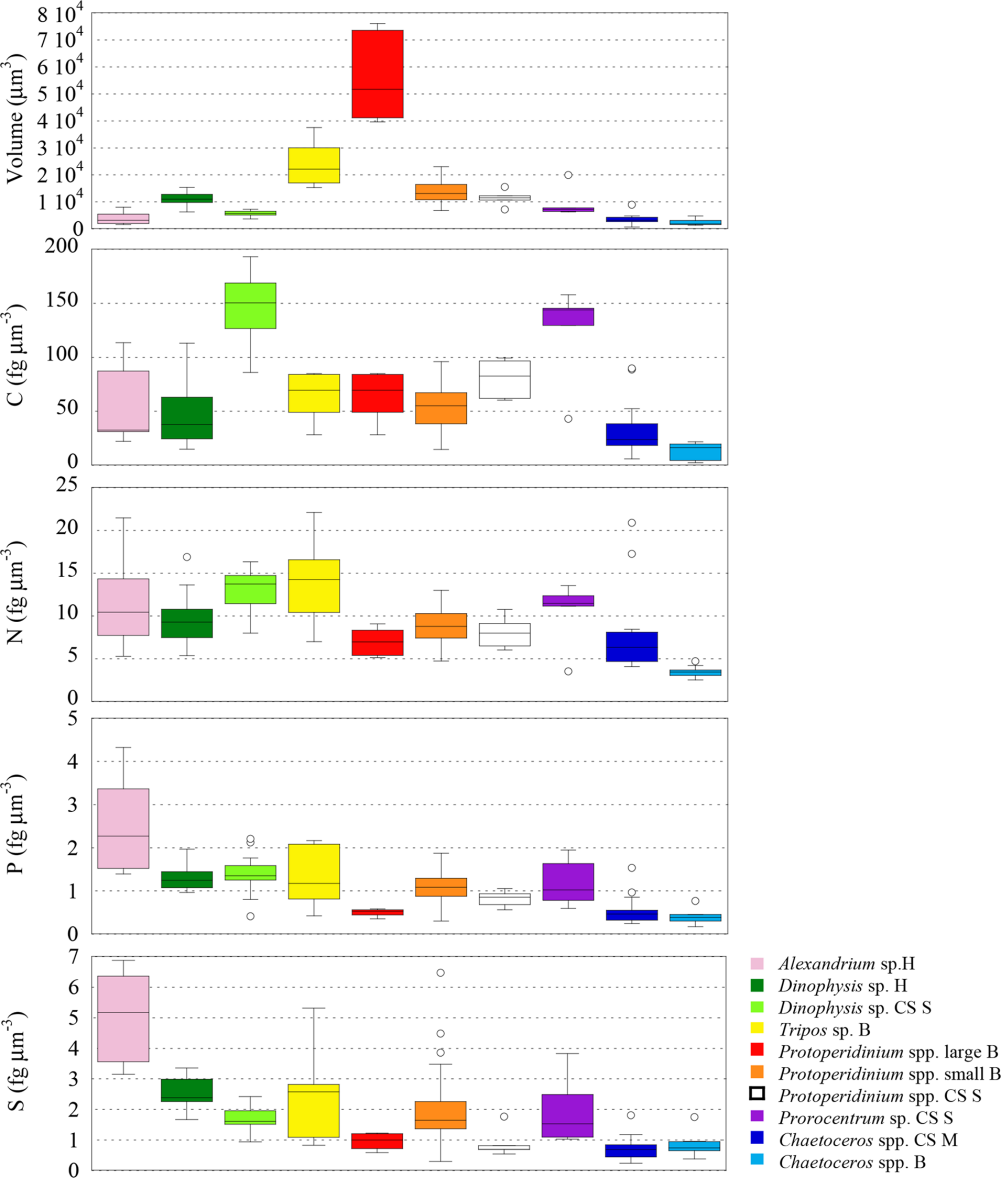
Box plots comparing cell volume (in μm^3^) and C, N, P and S elemental concentrations (in fg μm^-3^) of dinoflagellates and diatoms from the NW Mediterranean Sea. H: Harbour; B: Bay; CS S: Continental Shelf Stratified water column; CS M: Continental Shelf Mixed water column.

**Fig 7:**
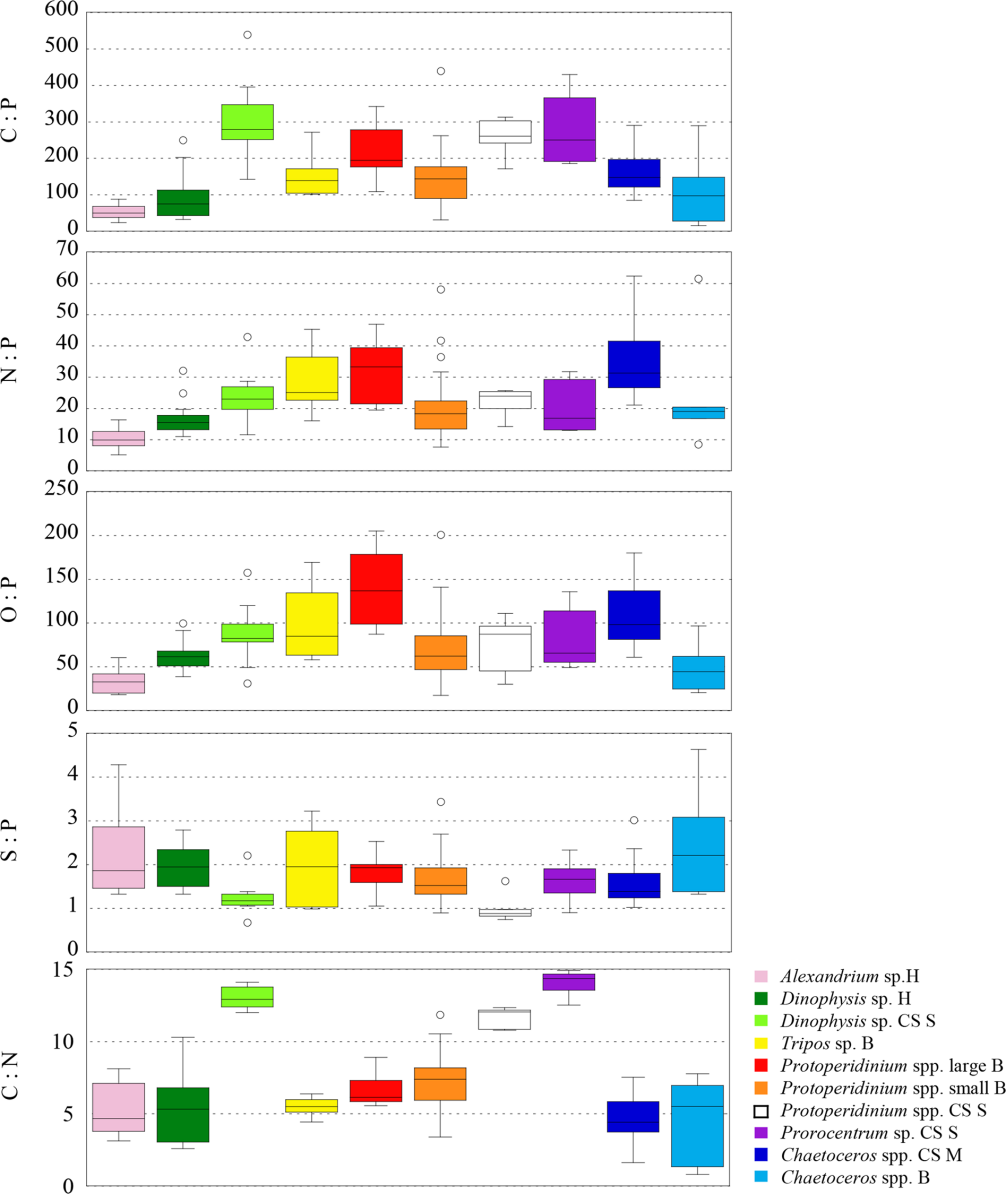
Box plots comparing different molar ratios (C:P, N:P, O:P, S:P, and C:N) of dinoflagellates and diatoms from the NW Mediterranean Sea. H: Harbour; B: Bay; CS S: Continental Shelf Stratified water column; CS M: Continental Shelf Mixed water column.

Dinoflagellates sampled in the Harbour and in the Bay had significant differences in sizes (*p* ≤ 0.005) (Fig 6), so we expected to find higher elemental concentrations in smaller cells (i.e. *A*. *minutum* in the Harbour and small *Protoperidinium* cells and *T*. *furca* in the Bay site). We found that P and S followed that trend (*p ≤* 0.01), but not C neither N. As a result, the N:P ratio was significantly different (*p* ≤ 0.01) between our dinoflagellates of different sizes sampled at the same site (Fig 7). Often, low N:P is explained by an accumulation of inorganic P storage products [91], however, in *A*. *minutum* cells low N:P could indicate a high investment in the growing machinery of cell [92], as would be expected from a fast growing red tide blooming phytoplankton. On the other hand, the lower N concentration (although not significant *p* = 0.017) in the small heterotrophic *Protoperidinium* compared to the mixotrophic *T*. *furca* could be reflecting extra N associated with photosynthetic pigments.

Dinoflagellates of similar sizes sampled from the CS Stratified site also showed inter-generic differences: C and N were significantly higher in *D*. cf. *punctata* compared to *Protoperidinium* (*p ≤* 0.01, Fig 6). The same trend, albeit not significant, was observed in the rest of elements, and thus the ratios C:P, N:P, O:P and S:P were not significantly different (*p* > 0.01). Only the C:N ratio was significantly different between *Protoperidinium* and the other two genera (*p ≤* 0.01, Fig 7).

These inter-generic differences highlight the variability in elemental concentration at the smallest level of observation. Thus, while the whole dataset, which includes a variety of species, sizes and environmental conditions representative of the NW Mediterranean Sea, shows the expected general trends (e.g. a scaling factor below 1 between C, N and P content *vs*. cell volume), environmental and phylogenetic differences can blur or even reverse these trends in small samples. This phenomenon is common in observations in nature when we go from large scale to micro or individual scale and are often the basis of contradictory conclusions in publications.

Intra-specific differences on elemental concentrations have been extensively studied in the laboratory using cultures of many different species [4]. However, field data is scarce because this comparison can only be done using single-cell techniques. For example, it has been demonstrated using SXRF that the quotas of P, S and trace elements in *Synechococcus* varied significantly between three different mesoscale eddies in the Sargasso Sea [93]. To our knowledge, our study is the first to report intra-specific comparison of the elemental composition of single microphytoplankton cells sampled from the field, under contrasting environmental conditions (Fig 1, Table 1), including C, N and O. Our sampling sites represent a gradient in nutrients availability and physical conditions: high nutrient concentrations and high stratification in the Harbour site, high nutrient concentrations and mixed water column in the CS Mixed site, and high stratification and low nutrient concentrations in the CS Stratified site. The Bay site represents an intermediate nutrient state between the last two (Table 1), however, Chla concentration was unusually high for the time of the year (more than 4 times higher than the average value for May for the last 5 years, with values closer to those found in winter (November to March) [24], when mixing with deeper water fertilizes the surface of the Catalan Sea), and was probably associated to a relatively recent nutrient input through a flood from the nearby river Tordera [94]. While the species assemblage varied accordingly among samples [21], few genera were common in two of the samples, giving us the chance to observe the effect of the environment on the elemental concentration and stoichiometry.

Two dinoflagellates genera (*D*. cf. *punctata* and *Protoperidinium* spp.) and one diatom (*Chaetoceros* spp.) were sampled in different sites. We found that, independently of changes in the size of dinoflagellates (*D*. cf. *punctata* were significantly lower (*p* ≤ 0.0001), but *Protoperidinium* spp. cells had similar (*p* = 0.20) size), cells collected in nutrient poor environments had significantly lower S and higher C concentration (*p* ≤ 0.01), while P concentration did not change (*p* > 0.05 to 0.23) (Table 2, Fig 6). N concentrations were higher (*p* ≤ 0.001) in *D*. cf. *punctata* from the nutrient rich Harbour, but were not different in *Protoperidinium* spp. (*p* = 0.41). This resulted in significantly higher C:P (*p* ≤ 0.01), C:N (*p* ≤ 0.001), and lower S:P (*p* ≤ 0.01) ratios in cells living in low nutrient waters, as well as larger N:P (*p* ≤ 0.01) in *D*. cf. *punctata*. In the case of the diatom *Chaetoceros* spp., with similar cell sizes in both sites (*p* = 0.12), we found larger C (*p* ≤ 0.01), N (*p* ≤ 0.0001) and Si concentrations (*p* ≤ 0.0001) in cells sampled from nutrient rich conditions (Fig 6). Like in dinoflagellates, there was no difference in the P concentration between the two groups (*p* = 0.35). Because changes in concentration of all elements were of the same sign, the stoichiometric ratios were not significantly affected, except for N:P (*p* < 0.01), and for Si:P (*p* ≤ 0.001). Larger C concentration in cells growing under rich nutrient conditions is opposite to what we found in dinoflagellates and what is known in cultured cells [4]. However, since the diatoms were not growing in such contrasting environments in terms of nutrients, as the dinoflagellates compared (nutrient rich environments -CS M or H- versus low nutrient environments -CS S-), our diatoms results could be reflecting the environmental history of the sampling sites, with elemental composition typical of nutrient-rich environments.

The plasticity of the C:N:P ratio of phytoplankton in the field and in laboratory cultures has been subject of interest to biochemists and physiologists for decades (e.g., [4]). In N-limited cultures, increases in C:N and decreases in N:P are usually observed. In P-limited cells, on the other hand, both C:P and N:P ratios increase. The changes observed in C:P and N:P ratios of the dinoflagellates in this study fit the paradigm of P-limited cells. It is interesting to remark that nutrients scarcity does not affect the P concentration in any of the genera compared (*Dinophysis*, *Protoperidinium* and *Chaetoceros*), suggesting that the concentration of P in these cells is already at the minimum critical level adapted to the low phosphate concentrations typically found in the Mediterranean Sea.

Our findings also show that significant differences in the elemental ratios and elemental concentrations exist not only between high taxonomic levels, but also at the genera level. In phytoplankton ecology, the occurrence of a relative high species diversity in a given phytoplankton assemblage under the same environmental conditions [95] usually has been explained as a combination of microenvironments or non-equilibrium states and different strategies of each species (e.g., [96, 97]). Different strategies may involve different physiologies and nutrient requirements, and this will necessarily lead to inter-generic and even inter-specific differences in the elemental composition and stoichiometry. Our results strengthen the idea that the stoichiometry of whole phytoplankton communities in the field are the result of biomass contribution, as well as of the phenotypic and genotypic characteristics of the present species, and improve the understanding and parametrization of variations in phytoplankton physiology, observed or modelled, in the marine system.

## Conclusions

To our knowledge, this study presents the first data of C, N, O, Mg, Si, P and S elemental concentration and stoichiometry of single dinoflagellate and diatom cells collected from the sea. These values have been obtained using X-ray microanalysis, the only single-cell method that can simultaneously identify and quantify all these elements in individual cells. We have validated this new methodology by putting the data obtained from individual cells from the NW Mediterranean Sea within the frame of historical data on phytoplankton elemental composition and stoichiometry. Our results indicate that, except for Si and O in diatoms, dinoflagellates are denser in all the elements compared to diatoms, and that the masses of C, N, Mg, P and S are not a constant fraction of cell volume but rather decrease with increasing cell volume. If these results turn out to be extrapolated beyond the limited set of species analysed important errors in the prediction of C, N, O, P and S from volume measurements may occur if the same conversion equations are used for both groups.

The elemental composition and stoichiometry of the cells analysed reflects the nutrient composition of deep Western Mediterranean Sea, a largely recognized P-limited system. Firstly, the N:P (slope ± standard error) for both dinoflagellates (24.6 ± 1.5) and diatoms (38.9 ± 3.0) is higher than the canonical 16:1 Redfield ratio, and closer to the nutrients ratio in deep NW Mediterranean waters (N:P = 20–23). Secondly, a comparison of the P concentration of our cells with other published results of bacterioplankton show that the P requirement is highest for bacterioplankton, followed by dinoflagellates, and lowest for diatoms. A low P-requirement represents a clear ecological advantage in this P-limited environment. And finally, the intra-generic comparison of cells sampled under different conditions show that there are changes in the C, N and S concentration between sampling sites, but not in the P concentration in any case, which suggests that the P quota of these cells is at the critical level, and determined by their phylogenetic biochemical characteristics. Consequently, and following Redfield's theory [67], the remineralization of the cells with high N:P, like those measured in this study, should be the source of the high N:P ratio observed in the deeper waters of the Western Mediterranean sea. Further XRMA single-cell analyses, not only of diatoms and dinoflagellates but also of other groups whose contribution to the export of organic material to deep waters is relevant, are needed to test this hypothesis.

## Supporting Information

S1 Table Elemental quota (pg cell^-1^) and volume (V, μm^3^ cell^-1^) of single dinoflagellates and diatom cells from the Catalan Sea (NW Mediterranean Sea). (XLS)

S2 Table Average ± standard deviation elemental concentrations (fg μm^-3^), dry weight (fg μm^-3^) and volume (V, μm^3^ cell^-1^) of alive dinoflagellate and diatom cells from the Catalan Sea. (DOC)

S3 Table Elemental ratios (mol:mol, mean ± standard deviation) of individual dinoflagellate and diatom cells from the Catalan Sea. Shown are the mean and standard deviation of cells of the same species or genera at each sampling site, which are characterized by different environmental conditions. Sites: H, Harbour; B, Bay; CS S, Continental Shelf Stratified; CS M, Continental Shelf Mixed. *n*: number of cells analysed.
(DOC)
