# Supplementary material for: Taxonomic and Environmental Variability in the Elemental Composition and Stoichiometry of Individual Dinoflagellate and Diatom Cells from the NW Mediterranean Sea
Source: PLoS One. 2016 Apr 25;11(4):e0154050. doi: 10.1371/journal.pone.0154050 (PMC4844132; doi:10.1371/journal.pone.0154050)
Supplement: S2 Table — (DOC) [file pone.0154050.s002.doc]

### S2 Table

### Average ± standard deviation elemental concentrations (fg µm^-3^) , dry weight (fg µm^-3^) and volume (V, **µm^3^ cell^-1^)** of dinoflagellate and diatom cells from the Catalan Sea.

| **Species** | **Site** | **n** | **C** | **N** | **O** | **Si** | **P** | **S** | **Dry weight** | **Volume** |
| --- | --- | --- | --- | --- | --- | --- | --- | --- | --- | --- |
| **Dinoflagellates** |  |  |  |  |  |  |  |  |  |  |
| ***Alexandrium* *minutum*** | H | 16 | 52.7 ± 32.2 | 11.3 ± 4.4 | 41.1 ± 15.8 | 2.8 ± 1.5 | 2.6 ± 1.0 | 5.1 ± 1.3 | 128.7 ± 55.3 | 3579.7 ± 2004.3 |
| ***Scrippsiella* sp.** | H | 4 | 28.1 ± 16.3 | 8.2 ± 2.0 | 25.9 ± 10.3 | 1.2 ± 0.2 | 1.5 ± 0.4 | 3.8 ± 0.8 | 77.5 ± 32.1 | 5617.0 ± 1759.5 |
| ***Dinophysis* cf. *punctata*** | H | 24 | 45.3 ± 28.5 | 9.4 ± 2.5 | 40.6 ± 8.6 | 1.5 ± 0.5 | 1.3 ± 0.3 | 2.5 ± 0.5 | 110.1 ± 38.7 | 11301.8 ± 2367.2 |
| ***Dinophysis* cf. *acuta*** | B | 1 | 62.6 | 7.6 | 35.2 | 0.9 | 0.5 | 0.6 | 116.0 | 33603.7 |
| ***Dinophysis* cf. *punctata*** | B | 1 | 19.5 | 5.4 | 22.8 | 0.4 | 0.9 | 1.9 | 55.4 | 9620.7 |
| ***Dinophysis* cf. *punctata*** | CS S | 13 | 147.0 ± 29.2 | 13.2 ± 2.5 | 56.8 ± 14.8 | 1.7 ± 0.7 | 1.4 ± 0.5 | 1.7 ± 0.4 | 240.4 ± 48.8 | 5692.8 ± 1030.7 |
| ***Neoceratium* *furca*** | B | 9 | 63.2 ± 22.1 | 13.5 ± 5.0 | 52.9 ± 20.8 | 1.4 ± 1.0 | 1.3 ± 0.6 | 2.4 ± 1.6 | 147.1 ± 54.0 | 24127.7 ± 8030.8 |
| ***Neoceratium fusus*** | CS S | 1 | 62.2 | 11.3 | 28.4 | nd | 0.7 | 1.3 | 114.4 | 12685.3 |
| ***Protoperidinium* spp. small** | B | 57 | 53.5 ± 18.2 | 8.7 ± 2.0 | 36.0 ± 11.2 | 1.0 ± 0.7 | 1.1 ± 0.4 | 1.9 ± 1.0 | 110.9 ± 31.6 | 13327.3 ± 3560.1 |
| ***Protoperidinium* spp. large** | B | 7 | 39.8 ± 9.2 | 7.0 ± 1.4 | 34.5 ± 6.7 | 0.5 ± 0.1 | 0.5 ± 0.1 | 0.9 ± 0.3 | 89.7 ± 16.5 | 54806.3 ± 14680.1 |
| ***Protoperidinium* spp.** | CS S | 6 | 80.4 ± 16.6 | 8.0 ± 1.7 | 30.7 ± 11.5 | 0.8 ± 0.3 | 0.8 ± 0.2 | 0.9 ± 0.4 | 133.1 ± 30.5 | 11378.9 ± 2680.2 |
| ***Prorocentrum* cf. *micans*** | B | 1 | 114.2 | 17.2 | 74.6 | 2.5 | 1.0 | 1.7 | 229.1 | 6647.6 |
| ***Prorocentrum* cf. *micans*** | CS S | 5 | 123.8 ± 46.2 | 10.4 ± 4.0 | 47.1 ± 17.3 | 0.8 ± 0.4 | 1.2 ± 0.6 | 2.0 ± 1.2 | 203.4 ± 74.5 | 9400.6 ± 5879.9 |
| **All dinoflagellates** |  | 145 | 63.5 ± 38.9 | 9.8 ± 3.3 | 40.1 ± 14.5 | 1.4 ± 1.0 | 1.3 ± 0.7 | 2.3 ± 1.4 | 129.3 ± 57.5 |  |
| **Diatoms** |  |  |  |  |  |  |  |  |  |  |
| ***Chaetoceros* spp.** | CS M | 19 | 31.2 ± 23.0 | 7.4 ± 4.4 | 27.1 ± 16.6 | 14.8 ± 8.9 | 0.5 **±** 0.3 | 0.8 ± 0.4 | 87.5 ± 56.4 | 3279.8 ± 1803.9 |
| ***Chaetoceros* spp.** | B | 10 | 13.5 ± 7.4 | 3.4 ± 0.6 | 9.2 ± 2.6 | 4.7 ± 2.4 | 0.4 ± 0.2 | 0.8 ± 0.4 | 36.1 ± 9.9 | 2322.3 ± 1244.5 |
| **Chaetoceros spp.** | CS S | 2 | 11.5 ± 0.6 | 4.3 ± 0.0 | 5.5 ± 0.7 | 2.4 ± 0.1 | 0.3 ± 0.1 | 0.3 ± 0.1 | 26.3 ± 0.3 | 2434.3 ± 110.0 |
| **Benthic diatom n.i.** | H | 1 | 19.4 | 7.3 | 34.6 | 21.5 | nd | nd | 87.2 | 5075.7 |
| **Centric diatom n.i.** | CS S | 1 | 6.3 | 22.3 | 159.0 | 114.2 | nd | nd | 306.4 | 8237.9 |
| **Pennate diatom n.i.** | B | 1 | 12.1 | 8.8 | 45.9 | 37.1 | nd | 0.6 | 109.6 | 3561.9 |
| ***Pseudo-nitzschia* spp.** | B | 3 | 12.7 ± 4.0 | 28.1 ± 2.2 | 39.9 ± 16.0 | 33.6 ± 4.5 | 1.5 ± 0.4 | 2.9 ± 0.9 | 129.3 ± 21.3 | 362.8 ± 55.0 |
| ***Pseudo-nitzschia* sp.** | CS S | 1 | 27.3 | 10.3 | 30.6 | 10.2 | 0.4 | 1.0 | 84.9 | 192.6 |
| ***Pleurosigma* sp.** | CS M | 3 | 26.5 ± 22.2 | 20.5 ± 1.0 | 134.4 ± 10.1 | 113.5 ± 14.5 | nd | nd | 300.6 ± 23.2 | 23353.8 ± 4387.1 |
| ***Rhizosolenia* sp.** | B | 3 | 5.8 ± 2.7 | 4.1 ± 0.3 | 6.4 ± 0.5 | 4.5 ± 1.2 | 0.3 ± 0.1 | 0.5 ± 0.3 | 23.3 ± 2.2 | 20852.9 ± 8054.6 |
| ***Thalassiosira* spp.** | CS M | 3 | 31.1 ± 3.6 | 18.9 ± 8.1 | 110.2 ± 39.5 | 105.9 ± 41.7 | 1.0 ± 0.3 | 0.9 ± 1.1 | 276.0 ± 89.3 | 3711.4 ± 1556.4 |
| **All diatoms** |  | 47 | 22.2 ± 18.7 | 9.5 ± 7.7 | 37.5 ± 38.4 | 27.4 ± 31.5 | 0.6 ± 0.4 | 0.8 ± 0.7 | 103.2 ± 83.2 |  |

n.i. : not identified. H: Harbour; B: Bay; CS S: Continental Shelf Stratified; CS M Continental Shelf Mixed. *n* = number of cells. nd = not detected.
