# Supplementary material for: Taxonomic and Environmental Variability in the Elemental Composition and Stoichiometry of Individual Dinoflagellate and Diatom Cells from the NW Mediterranean Sea
Source: PLoS One. 2016 Apr 25;11(4):e0154050. doi: 10.1371/journal.pone.0154050 (PMC4844132; doi:10.1371/journal.pone.0154050)
Supplement: S3 Table — Shown are the mean and standard deviation of cells of the same species or genera at each sampling site, which are characterized by different environmental conditions. Sites: H, Harbour; B, Bay; CS S, Continental Shelf Stratified; CS M, Continental Shelf Mixed. n: number of cells analysed. (DOC) [file pone.0154050.s003.doc]

### **S3 Table**

### **Elemental ratios (mol:mol, mean ± standard deviation) of individual dinoflagellate and diatom cells from the Catalan Sea.**

|  | **Site** | **n** | **C:P** | **N:P** | **O:P** | **Si:P** | **S:P** | **C:N** | **C:O** | **C:S** | **Si:N** | **Si:C** | **O:Si** |
| --- | --- | --- | --- | --- | --- | --- | --- | --- | --- | --- | --- | --- | --- |
| **Dinoflagellates** |  |  |  |  |  |  |  |  |  |  |  |  |  |
| ***Alexandrium* *minutum*** | H | 16 | 52.0 ± 19.5 | 10.1 ± 2.9 | 32.8 ± 12.9 | 1.3 ± 1.0 | 2.1 ± 0.8 | 5.2 ± 1.6 | 1.7 ± 0.7 | 28.1 ± 15.5 |  |  |  |
| ***Scrippsiella* sp.** | H | 4 | 45.0 ± 19.6 | 12.3 ± 2.8 | 32.8 ± 8.6 | 0.9 ± 0.2 | 2.5 ± 0.3 | 3.8 ± 1.4 | 1.4 ± 0.5 | 18.9 ± 9.3 |  |  |  |
| ***Dinophysis* cf. *punctata*** | H | 24 | 91.4 ± 56.6 | 16.4 ± 4.5 | 62.2 ± 14.2 | 1.3 ± 0.4 | 1.9 ± 0.4 | 5.3 ± 2.3 | 1.5 ± 0.8 | 52.9 ± 41.5 |  |  |  |
| ***Dinophysis* cf. *acuta*** | B | 1 | 331.3 | 34.5 | 139.8 | 2.1 | 1.2 | 9.6 | 2.4 | 265.1 |  |  |  |
| ***Dinophysis* cf. *punctata*** | B | 1 | 56.1 | 13.2 | 49.1 | 0.5 | 2.1 | 4.2 | 1.1 | 27.3 |  |  |  |
| ***Dinophysis* cf. *punctata*** | CS S | 13 | 299.6 ± 100.6 | 23.8 ± 7.8 | 96.3 ± 33.1 | 1.4 ± 0.5 | 1.2 ± 0.3 | 13.0 ± 0.7 | 3.6 ± 0.6 | 239.1 ± 38.5 |  |  |  |
| ***Neoceratium* *furca*** | B | 9 | 151.7 ± 54.3 | 27.8 ± 9.1 | 95.4 ± 37.8 | 1.2 ± 0.4 | 2.0 ± 0.8 | 5.5 ± 0.7 | 1.6 ± 0.3 | 95.7 ± 76.5 |  |  |  |
| ***Neoceratium* *fusus*** | CS S | 1 | 230.0 | 35.9 | 78.7 |  | 1.8 | 6.4 | 2.9 | 129.5 |  |  |  |
| ***Protoperidinium* spp. small** | B | 57 | 141.6 ± 70.8 | 19.6 ± 8.7 | 69.8 ± 33.9 | 1.4 ± 2.7 | 1.7 ± 0.5 | 7.1 ± 1.6 | 2.1 ± 0.6 | 94.1 ± 55.1 |  |  |  |
| ***Protoperidinium* spp. large** | B | 7 | 216.5 ± 75.5 | 32.3 ± 9.9 | 140.4 ± 42.2 | 1.2 ± 0.5 | 1.8 ± 0.5 | 6.7 ± 1.2 | 1.5 ± 0.3 | 121.9 ± 40.8 |  |  |  |
| ***Protoperidinium* spp.** | CS S | 6 | 258.2 ± 51.7 | 22.1 ± 4.5 | 76.0 ± 31.9 | 1.1 ± 0.3 | 1.0 ± 0.3 | 11.7 ± 0.7 | 3.8 ± 1.3 | 278.7 ± 84.6 |  |  |  |
| ***Prorocentrum* cf. *micans*** | B | 1 | 287.7 | 37.2 | 141.2 | 2.7 | 1.6 | 7.7 | 2.0 | 178.1 |  |  |  |
| ***Prorocentrum* cf. *micans*** | CS S | 5 | 284.4 ± 108.7 | 20.7 ± 9.1 | 83.8 ± 38.6 | 1.0 ± 0.6 | 1.6 ± 0.5 | 14.0 ± 1.0 | 3.5 ± 0.3 | 192.9 ± 99.1 |  |  |  |
| **All dinoflagellates** |  | 145 | 151.2 ± 100.1 | 19.6 ± 9.1 | 71.4 ± 37.4 | 1.3 ± 0.8 | 1.7 ± 0.6 | 7.3 ± 3.1 | 2.1 ± 1.0 | 104.9 ± 99.1 |  |  |  |
| **Diatoms** |  |  |  |  |  |  |  |  |  |  |  |  |  |
| ***Chaetoceros* spp.** | CS M | 19 | 163.5 ± 57.2 | 34.9 ± 12.0 | 109.7 ± 36.7 | 34.1 ± 10.8 | 1.5 ± 0.5 | 4.6 ± 1.4 | 1.5 ± 0.4 | 106.4 ± 34.2 | 0.97 ± 0.14 | 0.24 ± 0.13 | 3.31 ± 0.41 |
| ***Chaetoceros* spp.** | B | 10 | 110.3 ± 93.1 | 23.6 ± 17.2 | 48.3 ± 25.5 | 14.2 ± 7.5 | 2.5 ± 1.1 | 4.8 ± 2.7 | 2.0 ± 1,1 | 49.7 ± 31.5 | 0.74 ± 0.50 | 0.28 ± 0.31 | 3.97 ± 2.15 |
| **Chaetoceros spp.** | CS S | 2 | 107.8 ± 16.9 | 34.6 ± 7.1 | 39.6 ± 13.3 | 9.7 ± 2.5 | 1.0 ± 0.1 | 3.1 ± 0.2 | 2.8 ± 0.5 | 111.5 ± 25.1 | 0.28 ± 0.02 | 0.09 ± 0.01 | 4.04 ± 0.31 |
| **Benthic diatom n.i.** | H | 1 |  |  |  |  |  | 3.1 | 0.7 |  |  | 0.46 |  |
| **Centric diatom n.i.** | CS S | 1 |  |  |  |  |  | 0.3 | 0.1 |  |  | 7.60 |  |
| **Pennate diatom n.i.** | B | 1 |  |  |  |  |  | 1.6 | 0.4 | 53.3 |  | 1.29 |  |
| ***Pseudo-nitzschia* spp.** | B | 3 | 23.2 ± 9.9 | 43.6 ± 12.2 | 56.7 ± 30.6 | 25.8 ± 7.1 | 2.0 ± 0.9 | 0.5 ± 0.2 | 0.5 ± 0.2 | 12.3 ± 2.8 | 0.60 ± 0.07 | 1.19 ± 0.43 | 2.11 ± 0.90 |
| ***Pseudo-nitzschia* sp.** | CS S | 1 | 156.7 | 50.7 | 132.0 | 25.1 | 2.1 | 3.1 | 1.2 | 73.0 | 0.49 | 0.16 | 5.26 |
| ***Pleurosigma* sp.** | CS M | 3 |  |  |  |  |  | 1.5 ± 1.2 | 0.3 ± 0.2 |  | 2.74 ± 0.49 | 5.22 ± 6.74 | 2.37 ± 0.43 |
| ***Rhizosolenia* sp.** | B | 3 | 67.3 ± 57.1 | 37.0 ± 12.4 | 49.6 ± 15.9 | 19.2 ± 3.3 | 1.6 ± 0.5 | 1.7 ± 0.9 | 1.2 ± 0.6 | 54.2 ± 62.9 | 0.53 ± 0.13 | 0.39 ± 0.19 | 2.62 ± 0.42 |
| ***Thalassiosira* spp.** | CS M | 3 | 82.6 ± 21.9 | 39.8 ± 6.2 | 206.3 ± 16.6 | 111.9 ± 20.2 | 1.5 ± 0.8 | 2.2 ± 0.8 | 0.4 ± 0.1 | 78.1 ± 47.1 | 2.76 ± 0.40 | 1.43 ± 0.57 | 1.93 ± 0.30 |
| **All diatoms** |  | 47 | 124.5 ± 74.2 | 34.4 ± 13.9 | 93.6 ± 43.5 | 33.2 ± 12.8 | 1.7 ± 0.8 | 3.6 ± 2.2 | 1.3 ± 0.9 | 78.6 ± 45.3 | 1.09 ± 0.63 | 1.43 ± 2.23 | 3.22 ± 1.19 |

Shown are the mean and standard deviation of cells of the same species or genera at each sampling site, which are characterized by different environmental conditions. Sites: H, Harbour; B, Bay; CS S, Continental Shelf Stratified; CS M, Continental Shelf Mixed. *n*: number of cells analysed.
